# Relationship between childhood sexual abuse and attitudes toward premarital sexual permissiveness among middle school students in Luzhou, China

**DOI:** 10.1186/s12889-021-12490-1

**Published:** 2022-01-11

**Authors:** Zhang Rong, Zhang Wen, Liao Maoxu, Liu Ya, Fan Song, Wei Hui, Tan Xiaozhen, Ye Yunli

**Affiliations:** 1 School of Public Health, Southwest Medical University, Luzhou, China; 2 Shanghai Pudong New Area Center for Disease Control & Prevention, Shanghai, China

**Keywords:** Child sexual abuse, Premarital sexual permissiveness, Middle school students, China

## Abstract

**Background:**

In recent years, the number of adolescents engaging in premarital sex has increased, and an increasing number of childhood sexual abuse (CSA) cases have been reported in China. Many studies have indicated that CSA has a well-established association with risky sexual activities. However, only a limited number of studies have explored possible reasons for this association among middle school students, a population that may engage in premarital sex, which is critical for the development of interventions to prevent risky sexual behavior. Based on random samples of middle school students from a Chinese city, this article investigated the relationship between CSA and students’ premarital sexual permissiveness (PSP).

**Methods:**

In a cross-sectional study conducted between 2016 and 2017 in Luzhou, China, 2292 middle school and high school students aged 12–18 years were recruited by multistage random sampling. All students were administered anonymous questionnaires. Multiple linear regression and binary logistic regression analyses were conducted to analyze the relationship between CSA and PSP.

**Results:**

The prevalence of CSA was 15.4% (354/2292; 95% CI: 14–16.9%). A higher percentage of male respondents (18.2%) than female respondents (12.9%) had experienced CSA. A positive association between CSA and PSP was found among students. Respondents who had suffered CSA exhibited greater PSP, and this relationship was observed in the male sample, female sample and the total student sample in Luzhou (*β* = − 3.76, *P* < 0.05; *β* = − 2.79, *P* < 0.05; and *β* = − 2.84, *P* < 0.05, respectively). Respondents who had suffered CSA were also more likely to express a double standard about premarital sex (odds ratio [*OR*] =1.41, *P* < 0.05), especially among male students (odds ratio [*OR*] =1.63, *P* < 0.05).

**Conclusions:**

Sex differences in CSA and the relationship between CSA and PSP were significant among this large sample of middle school students in Luzhou (China). The findings suggest that experiencing CSA may be closely related to youth attitudes toward premarital sex, especially among males. Therefore, it is important to emphasize the prevention of CSA and provide adolescent reproductive health programs to reduce the impact of CSA on sexual cognition and attitude, prevent premarital sex and promote positive attitudes toward sexual equality for middle school students.

**Supplementary Information:**

The online version contains supplementary material available at 10.1186/s12889-021-12490-1.

## Background

In the last three decades, young people’s attitudes toward premarital sex in China have changed substantially, shifting from conservative to open. This phenomenon is highly related to the increased influence of Western culture after economic reform and opening up, the implementation of policies on late marriage and late childbearing in China, and early puberty in the general population. The prevalence of premarital sex has also risen dramatically [1, 2]. The health risks of teenagers’ premarital sex behaviors, such as HIV infection and other sexually transmitted infections (STIs) [3], unplanned pregnancy [4], and the risks associated with having multiple sexual partners [5], could lead to negative physical and psychological consequences and pose a serious threat to the healthy development of adolescents. Data on the prevalence of premarital sex among male adolescents in two other Asian cities are not optimistic [6], showing prevalences of 24.1% in Taipei, Taiwan, and 5.2% in Hanoi, Vietnam.

Youth have been identified as the population group that is the most vulnerable to unwanted pregnancy, HIV infections and STIs. A study on 4769 female undergraduates reported that 18% of the students had previously had sex and that the proportions of students with self-reported pregnancies and STIs were 17.5 and 20.4%, respectively [7]. A cross-sectional study including university students from 49 universities across 7 cities in China indicated that approximately one-third of unmarried female university students engaging in sexual intercourse had experienced unintended pregnancy [8]. Seventy-seven percent of newly diagnosed HIV/AIDS patients in China were students from colleges and universities, and the main transmission route was sexual contact [9].

The prevalence of childhood sexual abuse (CSA) is 6–18.7% in teenagers aged 18 and below in China [10–12]. However, CSA data may have been underreported in past studies, as an increasing number of CSA cases have been recently reported in Chinese media and professional journals. Chinese population studies have also found that CSA is related to early sexual debut among children and adolescents in rural areas [13–15]. Among the number of determinants of premarital sex [16–19], one important factor is CSA [20]. However, there is sparse existing evidence indicating whether CSA affects attitudes toward premarital sex in middle school students and whether CSA potentially affects the premarital sex behavior of university students and school dropouts.

Researchers have found that sexual attitudes are significantly positively correlated with sexual behaviors [21]. Research has shown that most students are young (16–18 years) at their first exposure to premarital sex [22], which indicates that the sexual attitudes of this age group could provide more insights into the correlations of sexual behaviors. Recent research on premarital sex has mainly focused on the determinants and health effects of sexual behaviors [3], and most of them recruited college students [23] or adults [19, 24, 25] as the subjects. Middle school students, as a population with a potential high risk of engaging in premarital sex, have rarely been included in existing studies. In addition, social cognitive theory (SCT) is widely used as a theoretical structure to explain individual behaviors as a result of three factors: environment, person, and behavior [26]. The model believes that every two out of the three factors can interact with each other and then influence the third factor; for example, human behaviors can be developed and modified by the direct experience of individuals and the structures of the environment [27, 28]. Therefore, we suspect that CSA as a direct personal experience may affect premarital sex. In addition, according to knowledge, attitude, belief, practice (KAP) theory, behavior and attitude are closely related. Therefore, it is suitable to use SCT to discuss the influence of CSA on the attitude of middle school students toward premarital sex.

Therefore, it is vital to understand the factors associated with attitudes toward premarital sexual behavior in adolescents. Additionally, the question of how CSA influences adolescents’ attitudes and beliefs about premarital sexual initiation in a society dominated by Confucian cultural norms has rarely been studied. The present study could support further efforts for the intervention and prevention of high-risk sexual behavior based on the following four major research objectives: (a) to understand attitudes toward premarital sex among middle school students; (b) to understand the prevalence of CSA among middle school students; (c) to explore the determinants of middle school students’ attitudes toward premarital sexual behavior; and (d) to determine whether CSA has an association with attitudes toward premarital sexual behavior and the strength of the association.

## Methods

### Study design and sample

The research data were extracted from a school-based comprehensive sex education program in Luzhou, Sichuan Province. Luzhou City is a third-tier city in China located in the south-central part of Sichuan Province. Its economic development level is representative of the vast majority of cities in central China. The data for this article were collected via a cross-sectional survey of 2396 students aged 12–18 years from eight different middle schools in urban and rural areas of Luzhou that was conducted by a team of researchers from Southwest Medical University. In this study, 2292 students were included in the analysis.

Multistage random sampling was used in the study. The seven administrative divisions of Luzhou City can be divided into three categories according to their economic status. Among the seven divisions, two (Jiangyang and Longmatan) have a good economy, three (Naxi, Luxian and Hejiang) have a fair economy, and two (Gulin and Xuyong) have a poor economy. With reference to the yearbook of middle school students’ statistics, the proportion of middle school students across the three different economic categories is 1:2:1. Therefore, in the first stage, the numbers of selected administrative divisions from individual economic categories are 1, 2 and 1. We randomly selected four administrative divisions (Longmatan, Luxian, Hejiang, and Gulin) from the seven divisions in Luzhou. In the second stage, two schools in each division, including one school in an urban area and one school in a rural area, were randomly selected. The number of students per class in public secondary schools is relatively constant (approximately 45 students) in Luzhou. In the third stage, two classes in each grade were selected. Thus, eight schools with an average of 300 individuals in each were selected. The “Sichuan Adolescent Sexual Health Questionnaire”, which has been published elsewhere [29], was adapted by the research teams for the survey. The survey was conducted by investigators with unified training from late Dec 2016 to early Jan 2017. Respondents independently completed the anonymous questionnaire with the guidance provided. This study received approval from the Medical Ethics Committee at Southwest Medical University and collaborating local organizations.

### Measures

#### Sociodemographic variables

The *socio*demographic variables in the analysis were separated into three sets. The first set of variables represented the characteristics of respondents, namely, sex and grade (middle school/high school). Sex was a self-reported dichotomous variable, as was grade. The second set of variables included rural/urban residence, self-reported economic status and one-child family. Economic status was self-reported according to three categories (low, middle, and high). The third set of variables concerned parents’ information and included living or deceased parents and father’s/mother’s educational level. Educational level was based on the highest level of education parents had completed. Additional variables included history of exposure to pornographic content on electronic media, parents’ or friends’ approval of premarital sex and history of dating or falling in love with somebody.

#### Gendered attitudes toward premarital sex

Premarital sexual permissiveness (PSP) scores were calculated based on modified Guttman scales with six items [30]; PSP scores indicate an individual’s approval of a sexual act (coitus) under different conditions (with a casual acquaintance, with a boyfriend/girlfriend, or with a fiancé and fiancée). One scale (the female scale) is composed of three items measuring the respondent’s perceived level of agreement with females initiating intimate behaviors (coitus) at various stages before marriage (with a stranger or with someone whom the person is not in love with, with an unattached boyfriend whom the person is in love with, or with a fiancé whom the person is engaged to), and the other scale (the male scale) is composed of three similar items that ask about appropriate behavior for males. A Likert scale was utilized for scoring. For each item, the possible responses were 1 = “Strongly Agree,” 2 = “Agree,” 3 = “Neutral,” 4 = “Disagree,” and 5 = “Strongly Disagree.” The scores ranged from 3 to 15 for each scale, with larger numbers indicating that respondents more strongly disapproved of intimate behaviors at the relevant stage. For convenience, we standardized the scores on a scale from 0 to 100. That is, the highest score of 15 corresponds to 100 points on the percentile system, so if the actual score of the student on the scale is x points, then the percentile scale score is equal to x*(100/15). Respondents with higher scores were considered more intolerant. The standardized Cronbach’s α coefficients for the two scales were 0.86 and 0.87. In addition, if respondents showed higher permissiveness regarding males’ premarital sexual behavior than that regarding females’ premarital sexual behavior, he or she was identified as having a double standard.

#### Adolescents’ attitudes toward gender roles

Studies have shown that gender role attitudes are also an important factor related to premarital sex [31]. The survey also collected data on respondents’ attitudes toward gender roles. Eleven items were included, and they were scored on a Likert scale where 1 = “Strongly Agree,” 2 = “Agree,” 3 = “Neutral,” 4 = “Disagree” and 5 = “Strongly Disagree.” The 11 items were as follows: (a) “Home affairs should be determined by the man in charge,” (b) “The most important task for a mother is to take care of her family and children,” (c) “The most important task for a father is to work hard to earn money,” (d) “Compared with boys, girls should start doing housework as young as possible,” (e) “Boys should receive a higher level of education than girls,” (f) “Parents should share the responsibility of raising children and doing housework,” (g) “Women can do what men do,” (h) “Contraception is mostly women’s responsibility,” (i) “For women, the greatest happiness is to find a good husband,” (j) “When jobs are scarce, men should be given priority over women,” and (k) “A woman can take the lead to express that she likes a man.” We reversed the scoring of items (f), (g) and (k) and summed the respondent’s scores to calculate the index. To obtain a clear view of the results, we again standardized the scores on a scale from 0 to 100. The Cronbach’s α coefficient was 0.686. Higher scores indicated more male chauvinism in the respondents’ conceptions and greater adherence to traditional gender roles.

#### Childhood sexual abuse

Referring to previous literature [29], we defined CSA as the experience of any of the following behaviors before age 18: (a) being the target of unwanted verbal flirtation (verbal sexual harassment); (b) being touched or hit in one’s chest or private parts; (c) being exposed to another naked person with the purpose of being made to feel inferior (forced exposure to pornography); (d) being forced to appear naked in front of someone else; (e) being forced to kiss another person, touch his or her body, take his or her clothes off and so on; and (f) being forced to engage in oral sex, anal sex, vaginal sex, genital friction or other sexual behaviors. Among them, items (a) ~ (d) were considered sexual harassment, item (e) was considered obscene, and item (f) was considered sexual assault.

### Analysis

We used EpiData Version 3.1 software (EpiData Association., DNK) and double entry to build the database. Subsequently, SPSS Statistics Version 22.0 (IBM, Inc., Amonk, NY) was used for data analysis. Percentages were used to describe the *socio*demographic characteristics of the objects. Second, the P-P graph showed that the distribution of PSP had a normal trend, which suggests the use of a parametric approach for data analysis. We applied a *t* test and analysis of variance to examine the differences in PSP scores of the respondents. The *χ*^2^ test was used to examine differences in respondents’ experience of CSA. The assumptions guiding the use of multiple linear regression were critically examined, and we ensured that none of these assumptions were violated. The independence index D-W statistics were 1.859, 1.907, and 1.925. There were no problems with collinearity. Normality and homogeneity of variance were basically satisfied, as shown in the residual graph (Supplementary Fig. 1, Additional file 1). Multiple linear regression was used to examine the influence of the *socio*demographic characteristics, CSA, and gender role attitudes of adolescents’ PSP in case of data compliance with assumptions. Logistic regression was used to explore the influencing factors of gendered double standards.

## Results

### Description of the sociodemographic characteristics of the respondents

A total of 2396 students were surveyed, and 2292 responses were valid. The *socio*demographic characteristics of the respondents are shown in Table 1. More than 50% of the students lived in rural areas. One-third of the respondents had previously dated or fallen in love with somebody. Nearly 70% of respondents reported that their parents and friends did not object to premarital sex, and 16% of the respondents had accessed pornographic content on electronic media (Table 1).

**Table 1:** Frequencies and percentages of *socio*demographic characteristics of the respondents (n (%))

*Socio*demographic characteristics	Frequency (Percent)
Sex	
Male	1091 (47.6)
Female	1201 (52.4)
Grade	
Middle school	1198 (52.3)
High school	1094 (47.7)
Left-behind student^a^	
Yes	1258 (57.2)
No	940 (42.8)
Residence^a^	
Rural	1274 (56.1)
Urban	998 (43.9)
Self-reported economic status^a^	
Low	627 (27.9)
Middle	1555 (69.1)
High	68 (3.0)
One-child family	
Yes	285 (12.4)
No	2007 (87.6)
Divorced parents	
Yes	216 (9.4)
No	2076 (90.6)
Living or deceased parents	
At least one parent deceased	106 (4.6)
No parent deceased	2186 (95.4)
Father’s education level^a^	
Primary school or below	715 (34.5)
Middle school	1057 (51.0)
High school and above	300 (14.5)
Mother’s education level^a^	
Primary school or below	1040 (51.8)
Middle school	742 (36.9)
High school and above	227 (11.3)
History of dating or falling in love with somebody^a^	
Yes	724 (33.2)
No	1460 (66.9)
Friends’ approval of premarital sex	
Yes and other responses	1785 (77.9)
No	507 (22.1)
Parents’ approval of premarital sex	
Yes and other responses	1672 (73.0)
No	620 (27.1)
History of exposure to pornographic content on electronic media	
Yes	367 (16.0)
No	1925 (84.0)

^a^Missing values

### Premarital sexual permissiveness

The *t* test and variance analysis show significant differences in PSP according to sex, grade, place of residence, self-reported economic status, one-child family status of the respondents, education level of the father and mother, a history of dating or falling in love with somebody, approval from friends and parents regarding premarital sex, a history of exposure to pornographic content on electronic media and experience of CSA. Both male and female respondents reported that males would have greater PSP than females. High school students expressed more permissiveness than middle school students. The following students were more permissive: students who resided in rural areas, had lower self-reported economic status, were members of one-child families, had previously dated or fallen in love with somebody, had friends and parents who approved of premarital sex, had previously been exposed to pornographic content on electronic media or had ever experienced CSA (Table 2).

**Table 2:** Respondents’ mean premarital sexual permissiveness scores, grouped by *socio*demographic characteristics (Total = 2292, Male = 1091, Female = 1201)

*Socio*demographic characteristics	Male & female scales			Male scale			Female scale		
	Total	Male	Female	Total	Male	Female	Total	Male	Female
Sex									
Male	80.29^**^	–	–	79.98^**^	–	–	80.61^**^	–	–
Female	90.36	–	–	90.30	–	–	90.42	–	–
Grade									
Middle school	87.84^**^	84.30^**^	91.12^*^	87.55^*^	83.97^**^	90.90	88.08^**^	84.60^**^	91.30^*^
High school	83.18	75.84	89.57	83.10	75.54	89.69	83.33	76.17	89.50
Left-behind student									
Yes	86.59^*^	80.58	91.52^**^	86.51^*^	80.38	91.56^**^	86.75^*^	80.91	91.51^**^
No	84.41	80.21	88.71	84.14	79.87	88.56	84.58	80.46	88.80
Residence									
Rural	86.65^**^	80.71	91.33^**^	86.48^**^	80.42	91.28^*^	86.85^**^	81.07	91.35^**^
Urban	84.25	79.87	88.93	84.01	79.54	88.85	84.45	80.15	89.06
Self-reported economic status									
Low	85.11^*^	79.69	90.69	84.78^*^	79.05	90.69^*^	85.66^*^	80.62	90.78
Middle	85.87	80.49	90.33	85.91	80.29	90.29	85.91	80.59	90.33
High	80.98^b^	78.97	85.44	80.98^a^	79.36	84.56^a^	81.07^a,b^	78.57	86.33
One-child family									
Yes	80.71^**^	76.03^*^	86.37^**^	79.93^**^	75.25^**^	85.65^**^	81.57^**^	77.00^*^	87.09^**^
No	86.30	81.01	90.84	86.18	80.79	90.86	86.39	81.22	90.82
Divorced parents									
Yes	84.06	78.44	88.76	83.74	78.06	88.45	84.56	78.99	89.19
No	85.75	80.47	90.53	85.57	80.17	90.50	85.91	80.77	90.55
Living or deceased parents									
At least one parent deceased	86.50	82.89	89.45	86.27	82.81	89.01	86.87	82.96	90.06
No	85.55	80.17	90.40	85.36	79.85	90.37	85.73	80.50	90.44
Father’s education level									
Primary school or below	86.28^**^	79.89	90.94^*^	86.11^**^	79.27	91.14^*^	86.52^**^	80.51	90.83^*^
Middle school	85.72	79.80	90.80	85.48	79.58	90.58	85.90	80.10	90.91
High school and above	82.00^ab^	79.08	86.95^ab^	82.20^ab^	79.42	86.98^ab^	81.75^ab^	78.68	86.91^ab^
Mother’s education level									
Primary school or below	86.48^**^	80.20	91.17^**^	86.27^*^	79.63	91.28^**^	86.64^**^	80.69	91.07^*^
Middle school	84.51^a^	78.74	90.23	84.37^a^	78.71	90.06	84.70^a^	78.95	90.38
High school and above	82.26^a^	78.52	86.38^ab^	82.03^a^	78.29	86.16^ab^	82.50^a^	78.75	86.60^ab^
History of dating or falling in love with somebody									
Yes	80.22^**^	73.76^**^	87.38^**^	80.14^**^	73.75^**^	87.33^**^	80.35^**^	73.90^**^	87.47^**^
No	88.43	84.28	91.85	88.25	83.97	91.79	88.59	84.58	91.89
Friends’ approval of premarital sex									
Yes and other responses	83.74^**^	79.21^**^	88.71^**^	83.46^**^	78.88^**^	88.91^**^	84.02^**^	79.55^**^	88.53^**^
No	92.09	86.92	94.29	92.19	86.76	93.97	91.92	87.08	94.51
Parents’ approval of premarital sex									
Yes and other responses	83.78^**^	79.34^**^	89.01^**^	83.45^**^	78.92^**^	88.82^**^	84.09^**^	79.74^*^	89.22^**^
No	90.41	84.91	92.67	90.58	85.18	92.85	90.24	84.86	92.46
History of exposure to pornographic content on electronic media									
Yes	74.44^**^	72.13^**^	86.26^*^	74.47^**^	72.02^**^	86.90^*^	74.47^**^	72.29^**^	85.73^*^
No	87.70	83.48	90.57	87.47	83.08	90.48	87.93	83.88	90.66
History of CSA									
Yes	80.34^**^	74.52^**^	87.61^*^	80.16^**^	74.01^**^	87.87^*^	80.52^**^	74.91^**^	87.47^*^
No	86.53	81.54	90.76	86.34	81.27	90.66	86.73	81.85	90.86

The test level is α = 0.05; “a” indicates that the difference compared with the first group is statistically significant, and “b” indicates that the difference compared with the second group is statistically significant

^*^P < 0.05

^**^P < 0.001

### Respondents’ experience of childhood sexual abuse

The prevalence of CSA was 15.4% (354/2292; 95% CI: 14–16.9%). The prevalence in males was higher than that in females (*P* < 0.001). The prevalence of CSA was different across different self-reported economic statuses (*P* < 0.05). Based on multiple comparisons, both male and female respondents reported that students with low self-reported economic status had more PSP than students with a middle self-reported economic status. Youth with a history of exposure to pornographic content on electronic media or a history of dating or falling in love with somebody had experienced a higher prevalence of CSA than others (*P* < 0.001). Female students whose parents did not approve of premarital sex had a lower rate of CSA (Table 3).

**Table 3:** Quantity (prevalence) of respondents with a history of CSA, grouped by *socio*demographic characteristics

Self-reported economic status characteristics	Total (*n* = 2292)	Male (*n* = 1091)	Female (*n* = 1201)
Sex			
Male	199 (18.2)^**^	–	–
Female	155 (12.9)	–	–
Grade			
Middle school	172 (14.4)	95 (16.3)	77 (12.5)
High school	182 (16.6)	104 (20.4)	78 (13.3)
Left-behind student			
Yes	196 (15.6)	99 (17.4)	97 (14.1)
No	140 (14.9)	87 (18.2)	53 (11.5)
Residence			
Rural	190 (14.9)	103 (18.3)	87 (12.3)
Urban	160 (16.0)	94 (18.2)	66 (13.8)
Self-reported economic status			
Low	126 (20.1)^*^	71 (22.4)^*^	55 (17.7)^*^
Middle	210 (13.5)^a^	114 (16.1)^a^	96 (11.4)^a^
High	12 (17.7)	10 (22.2)	2 (8.7)
One-child family			
Yes	34 (11.9)	22 (13.8)	12 (9.5)
No	320 (15.9)	177 (19.0)	143 (13.3)
Divorced parents			
Yes	31 (14.4)	18 (18.4)	13 (11.0)
No	323 (15.6)	181 (18.2)	142 (13.1)
Living or deceased parents			
At least one parent deceased	9 (8.5) ^*^	4 (8.5)	5 (8.5)
No	345 (15.8)	195 (18.7)	150 (13.1)
Father’s education level			
Primary school or below	111 (15.5)	54 (18.0)	57 (13.7)
Middle school	162 (15.3)	93 (18.8)	69 (12. 3)
High school and above	51 (17.0)	37 (19.7)	14 (12.5)
Mother’s education level			
Primary school or below	165 (15.9)	86 (19.3)	79 (13.3)
Middle school	116 (15.6)	70 (18.7)	46 (12.5)
High school and above	34 (15.0)	20 (17.0)	14 (12.8)
History of dating or falling in love with somebody			
Yes	209 (28.9) ^**^	117 (30.5) ^**^	92 (27.1) ^**^
No	125 (8.6)	73 (11.1)	52 (6.5)
Friends’ approval of premarital sex			
Yes and other responses	288 (16.1)	176 (18.7)	112 (13.3)
No	66 (13.0)	23 (15.3)	43 (12.0)
Parents’ approval of premarital sex			
Yes and other responses	254 (15.2)	169 (18.6)	85 (11.1) *
No	100 (16.1)	30 (16.4)	70 (16.0)
History of exposure to pornographic content on electronic media			
Yes	114 (31.1) ^**^	91 (29.7) ^**^	23 (37.7) ^**^
No	240 (12.5)	108 (13.8)	132 (11.6)

The test level is α = 0.05; “a” indicates that the difference compared with the first group is statistically significant, and “b” indicates that the difference compared with the second group is statistically significant

^*^P < 0.05

^**^P < 0.001

### Relationship between childhood sexual abuse and premarital sexual permissiveness

In the multivariate analysis, we used male respondents’ attitudes toward male premarital sex and female respondents’ attitudes toward female premarital sexual behavior as indicators of male and female respondents’ attitudes, respectively. Among all students, those who had experienced CSA were more permissive about premarital sex (*β* = − 2.84, *P* < 0.05). This relationship was also found among both the male and female student samples (*β* = − 3.76 and *P* < 0.05 for the male student sample and *β* = − 2.79 and *P* < 0.05 for the female student sample). Students who were in high school, were members of a one-child family, were not left-behind students, had lower scores for gender role attitudes (less traditional gender norms), had previously been exposed to pornographic content on electronic media, had previously dated or fallen in love with somebody, and had friends and parents who approved of premarital sex showed greater permissiveness. In addition, girls reported less PSP than boys (*β* = 6.18, *P* < 0.001) (Table 4).

**Table 4:** Coefficients (95% confidence intervals for *β*) from the stepwise linear regression model assessing the influencing factors of premarital sexual permissiveness, grouped by sex

Variables	Total (*n* = 2292)	Male (*n* = 1091)	Female (*n* = 1201)
Sex (ref: male)	6.18 (4.56 ~ 7.80)^**^	–	–
Grade (ref: middle school)	−2.49 (− 3.99 ~ − 0.98)^*^	−5.57 (−8.28 ~ − 2.87)^**^	–
One-child family (ref: no)	− 3.95 (−6.02 ~ − 1.88)^**^	−4.55 (− 7.93 ~ − 1.18)^*^	− 3.56 (− 6.06 ~ − 1.05)^*^
Left-behind student (ref: no)	1.47 (0.05 ~ 2.89)^*^	–	2.87 (1.27 ~ 4.47)^**^
Gender role attitudes	0.17 (0.10 ~ 0.24)^**^	0.19 (0.07 ~ 0.31)^*^	0.10 (0.02 ~ 0.18)^*^
History of exposure to pornographic content on electronic media (ref: no)	−6.88 (− 8.90 ~ − 4.86)^**^	− 6.94 (−9.90 ~ − 3.99)^**^	–
History of dating or falling in love with somebody (ref: no)	− 4.66 (− 6.24 ~ − 3.07)^**^	−5.40 (− 8.18 ~ − 2.61)^**^	−3.87 (− 5.59 ~ − 2.15)^**^
Friends’ approval of premarital sex (ref: no)	−4.51 (− 6.28 ~ − 2.74)^**^	− 5.30 (− 8.97 ~ − 1.63)*	−3.43 (− 5.20 ~ − 1.65)^**^
Parents’ approval of premarital sex (ref: no)	− 3.06 (− 4.70 ~ − 1.42)^**^	−4.77 (− 8.12 ~ − 1.42)^*^	−1.81 (− 3.48 ~ − 0.14)^*^
CSA (ref: no)	−2.84 (− 4.81 ~ − 0.88)^*^	− 3.76 (− 7.00 ~ − 0.52)^*^	− 2.79 (− 5.18 ~ − 0.40)^*^
Constant	72.98 (67.46 ~ 78.51)	83.53 (74.52 ~ 92.55)	85.61 (79.08 ~ 92.13)

^*^P < 0.05

^**^P < 0.001

Those who had experienced CSA were more likely to express double standards about premarital sex (odds ratio [*OR*] =1.41, *P* < 0.05), especially among male students (odds ratio [*OR*] =1.63, *P* < 0.05) (Table 5). Generally, male students who had lower gender role attitude scores (less traditional gender norms), had suffered CSA, or had previously been exposed to pornographic content on electronic media were more likely to express double standards. Female students who had previously dated or fallen in love with somebody or had lower gender role attitude scores (less traditional gender norms) were also more likely to express double standards about PSP (Table 5).

**Table 5:** Odds ratio (95% confidence interval) from the logistic regression model assessing the influencing factors of holding a double standard about premarital sexual permissiveness

Variables	MODEL 1	MODEL 2	MODEL 3
Sex (ref: male)	–	–	0.65 (0.52 ~ 0.82)^*^
History of dating or falling love with somebody (ref: no)	–	1.79 (1.29 ~ 2.49)^*^	1.38 (1.09 ~ 1.75)^*^
Gender role attitudes	0.98 (0.96 ~ 0.99)^*^	0.98 (0.97 ~ 1)^*^	0.98 (0.97 ~ 0.99)^*^
CSA (ref: no)	1.63 (1.11 ~ 2.39)^*^	–	1.41 (1.05 ~ 1.88)^*^
History of exposure to pornographic content on electronic media (ref: no)	1.59 (1.13 ~ 2.23)^*^	–	1.36 (1.01 ~ 1.83)^*^
Constant	3.73	1.97	3.94

Note: MODEL 1: male middle school students with a double standard

MODEL 2: female middle school students with a double standard

MODEL 3: middle school students with a double standard

All three models were conducted by controlling the covariates: grade, divorced parents, living or deceased parents, self-reported economic status, one-child family, father’s and mother’s education level, residence, and left-behind status of middle school students. The covariate sex was controlled only for model 3. *P < 0.05

## Discussion

Attitude determines behavior, as shown by KAP theory. The middle school period is a crucial period for the formation of adolescents’ thoughts and values. Students’ attitudes toward premarital sex in this period influence their sexual behavior patterns in college and even in adulthood. A positive association between CSA and PSP was found among middle school students in Luzhou. The findings of this study provide a new perspective and strategy for the intervention of adolescent premarital sex.

Our findings suggested obvious sex differences in permissive sexual attitudes. The results of the multiple linear regression models demonstrated that male middle school students reported more permissive sexual attitudes than female middle school students. This difference was consistent in the male, female and general samples, as we expected and in line with empirical findings stressing that adolescents’ sexuality develops, at least on certain dimensions, in a gender-specific way [13, 32]. Several explanations for this difference have been proposed. First, gendered attitudes toward PSP may be due to the different restrictions imposed on male and female sexual behaviors under Confucianist cultural norms. Confucianism, collectivism, male chauvinism, and asceticism are important features of China’s culture, and these aspects have played an important role in shaping the beliefs and behaviors of much of the population [33]. For example, a young girl is not encouraged to take the initiative in expressing her affections to a man; instead, a young man is encouraged to take the initiative. Sexual activity is taboo and forbidden before marriage. Second, feelings of shame or guilt may cause female middle school students to underreport their actual attitudes [32]. Third, a lack of sex education at all ages creates a barrier for students’ understanding of different forms of CSA, and males are exposed to pornography earlier and more easily than females [34, 35].

The prevalence of CSA among Luzhou middle school students was higher than that reported in prior meta-analyses [36] and another study, where the overall lifetime prevalence of CSA was 8% [37]. One possible explanation is that studies on CSA usually use retrospective self-reporting methods, which inherently involve some recall bias. Such bias increases as respondents age [38]. Since our subjects were middle school students, the recall bias should be smaller, as the time since the experience of CSA was shorter. Alternatively, the observed prevalence may suggest that the total prevalence of CSA is increasing in China. This study revealed that the male prevalence of CSA was higher than that of females (*P* < 0.001), which is consistent with the findings that boys were more likely to report CSA than girls [37]. The higher prevalence of CSA among male students in the study may be due to the broad definitions of sexual harassment, which include forced exposure to pornography and verbal sexual harassment. Male students may have more chances of being exposed to sexual harassment than female students, which may lead to a high prevalence of CSA among male students. In the context of general Chinese culture and the one-child policy, CSA has been considered too sensitive to discuss publicly [37]. School-aged girls are probably more likely to underreport their experiences. Parental supervision of children, especially girls, is generally strict due to the moral value placed on women’s chastity in Chinese culture, which may protect Chinese girls from experiencing CSA [39]. When the issue of CSA in China has been discussed in the past, attention has mainly been focused on females as the key at-risk population. However, this study suggests that the CSA prevalence among male adolescent students can be quite alarming, and more prevention should be considered.

Similar to other studies [11, 40], we found numerous factors related to attitudes toward premarital sex. After controlling for sex, grade, one-child family status, left-behind status of middle school students, gender role score, a history of exposure to pornographic content on electronic media, a history of dating or falling in love with somebody, and parents’ and friends’ attitudes toward premarital sex, we found that among the general population, those who had CSA experience had more open attitudes toward premarital sex. The stratified analysis also showed that middle school students who had CSA experience were more open to premarital sex in the male and total student samples. According to social cognitive theory (SCT), under the combined effect of direct experience and result expectations, sexually curious middle school students who have experienced SCA will be more open to premarital sex than others. Among them, CSA can be considered a direct experience process in childhood, and the less serious health consequences are the positive outcome expectations. A previous study also suggested that those with a history of CSA had greater motives to use sex to cope with negative impacts and engaged in more risky sexual behaviors. This relationship was observed among male youth but not among female youth [40], which is similar to our finding. Based on the notion that masculinity norms (gender-based, heterocentric stereotypes) might drive increased sexual risk taking among males with a history of CSA compared to that in males without CSA experience, males with a history of CSA may utilize sex to manage distress and gain social approval [41]. Due to the male-dominated sexual culture in traditional Chinese culture, males’ openness about sexual behaviors may greatly affect the premarital sexual behaviors of teenagers. However, in our study, a relationship between CSA experience and PSP was also found among female students. The results also indicated that female students expressed a desire for more gender equality in relationships, as more males than females in the sample held double standards about PSP. Therefore, women who suffer CSA will inevitably suffer gender inequality and deal with the negative impact of CSA experience, which can potentially manifest as greater openness to premarital sex. Therefore, the intervention of premarital sexual behaviors of middle school students should consider CSA experience, regardless of whether the intervention is with male or female groups.

Double standards (students being more permissive about premarital sex initiated by males than by females) were common among both male and female students but more pronounced among males, who are generally more open toward premarital sex than women because of cultural norms of masculinity. Meanwhile, male high school students who had experienced CSA were also more likely to hold double standards than other students. This finding could be explained by the fact that CSA experience was reported to be higher in male high school students who were potentially more open about premarital sex. The CSA experience rate was also reported to be higher in males than in females.

This study had some limitations. First, although the study involved a large-scale middle school student sample in Luzhou city, Sichuan Province, which is a typical city that is representative of third-tier cities in China, the sample was drawn from only one city. Thus, the sample was representative only of middle school students with similar socioeconomic statuses. The generalizability of the findings to populations in other cities, especially those with other socioeconomic contexts, is limited. Second, although we used self-administered anonymous questionnaires, underreporting bias might still exist because the topic of premarital sex is sensitive and unacceptable to talk about for most Chinese middle school students in Chinese cultural settings. However, we explained the purpose and significance of the survey to the respondents before the survey, and respondents completed the survey independently and anonymously, with students seated the same distance from students that they are during exams to safeguard the respondents’ privacy and minimize bias. Third, in our study, we found that middle school students with CSA experience held more open attitudes toward premarital sex than those without CSA experience. However, as with many other self-reporting surveys, there may have been a gap between the reports and reality. Hence, our findings on the effect of CSA experience on sexual attitudes may be tentative and need to be replicated with more empirical studies. Fourth, the definition of CSA in this study, which considers culture and population adaptation, was defined based on relevant research at home and abroad. However, this definition is inconsistent with the measurement of CSA used by other researchers and results in a decrease in comparability of research results. Fifth, in this study, we adopted a self-reporting method to collect information about the economic status of the respondents. This subjective reporting method may misclassify respondents into the wrong category. Furthermore, because the sample was cross-sectional, our findings on the effect of CSA on premarital sexual attitudes showed a possible association but not a causal relationship. We suggest that in future studies, longitudinal approaches should be utilized to better understand the causality between CSA experience and PSP.

Gender differences in CSA and the relationship between CSA and PSP were significant among middle school students in Luzhou. Child sex abuse may highly influence victims’ attitudes toward premarital sex in their adolescent life, especially for male victims. Therefore, awareness to help prevent child sex abuse must be raised for families, schools and communities. In addition, child sex abuse preventive programs are crucial to reduce the risk of child sex abuse, promote sexual equality and develop positive attitudes toward premarital sex. Preventive programs can be conducted by both family and school interventions in various forms and settings, such as sex education in communities, kindergartens, and primary schools. To enhance the performance of sex education, relevant stakeholders should become involved, and more resources should be invested in to strengthen teacher training programs and facilitate active cooperation between families and schools. It is crucial to employ various measures to reduce the risk of child sex abuse, promote positive attitudes toward premarital sex and alleviate the impact of child sex abuse on sexual cognition and attitudes.

## Conclusions

The findings showed that gender differences in CSA and the relationship between CSA and PSP were significant among middle school students in Luzhou, China. CSA experience is a significant factor in shaping adolescent sexual attitudes, especially among males. Some new directions for future research are indicated in the present study. The CSA experience of males should be given the same or even greater attention than that of females. Further research on adolescent sexual and reproductive outcomes should include the elements of CSA experience and examine differences and similarities between genders. In addition, future longitudinal studies on the premarital sexual behavior of adolescents need to further verify the relationship between CSA experience and attitudes toward premarital sex, as the specific pathway of this influence is not yet clear.

## Supplementary Information

**Additional file 1: Figure 1.** Residual graph of total, male and female students.

## Data Availability

The datasets used during the current study are available from the corresponding author upon reasonable request.

## Abbreviations

CSA: Childhood sexual abuse
STIs: Sexually transmitted infections
PSP: Premarital sexual permissiveness
SCT: Social cognitive theory
KAP: Knowledge, attitude, belief, practice theory
