# Supplementary figures and images for: Relationship between childhood sexual abuse and attitudes toward premarital sexual permissiveness among middle school students in Luzhou, China
Source: BMC Public Health. 2022 Jan 11;22:71. doi: 10.1186/s12889-021-12490-1 (PMC8753910; doi:10.1186/s12889-021-12490-1)

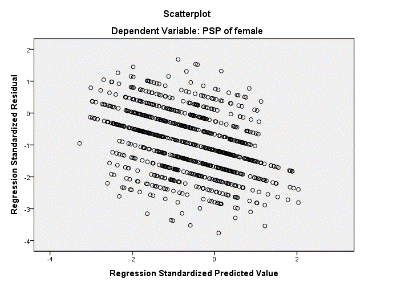

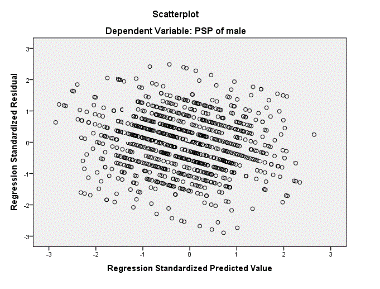


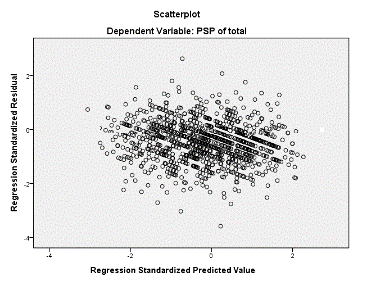


a b c

Figure 1. Residual graph of total, male and female students

Supplement: Supplementary file 1 — Additional file 1: Figure 1. Residual graph of total, male and female students. [file 12889_2021_12490_MOESM1_ESM.docx]
